# Supplementary material for: Effect of prolonged sitting immobility on shear wave velocity of the lower leg muscles in healthy adults: A proof-of-concept study
Source: PLoS One. 2021 May 10;16(5):e0251532. doi: 10.1371/journal.pone.0251532 (PMC8109794; doi:10.1371/journal.pone.0251532)
Supplement: S1 Table — (DOCX) [file pone.0251532.s001.docx]

**S1 Table. Shear wave velocity of the lower leg muscles (n=24).**

| Lateral gastrocnemius | |  |  |  |
| --- | --- | --- | --- | --- |
|  | Start of sitting | 60 min | 120 min | After leg raise |
| Mean | 1.52 | 1.58 | 1.70 | 1.52 |
| SD | 0.06 | 0.06 | 0.10 | 0.07 |
| SE | 0.01 | 0.01 | 0.02 | 0.01 |
| Min | 1.40 | 1.41 | 1.43 | 1.39 |
| Max | 1.68 | 1.72 | 1.90 | 1.63 |
| Median | 1.53 | 1.57 | 1.70 | 1.53 |
| CV (%) | 4.08 | 4.04 | 5.87 | 4.75 |

| Medial Gastrocnemius | |  |  |  |
| --- | --- | --- | --- | --- |
|  | Start of sitting | 60 min | 120 min | After leg raise |
| Mean | 1.66 | 1.70 | 1.83 | 1.59 |
| SD | 0.10 | 0.09 | 0.11 | 0.07 |
| SE | 0.02 | 0.02 | 0.02 | 0.02 |
| Min | 1.51 | 1.56 | 1.66 | 1.44 |
| Max | 1.89 | 1.92 | 2.07 | 1.73 |
| Median | 1.65 | 1.69 | 1.81 | 1.59 |
| CV (%) | 6.19 | 5.52 | 6.17 | 4.59 |

| Soleus |  |  |  |  |
| --- | --- | --- | --- | --- |
|  | Start of sitting | 60 min | 120 min | After leg raise |
| Mean | 1.60 | 1.69 | 1.89 | 1.55 |
| SD | 0.15 | 0.15 | 0.17 | 0.11 |
| SE | 0.03 | 0.03 | 0.03 | 0.02 |
| Min | 1.31 | 1.47 | 1.62 | 1.32 |
| Max | 2.04 | 2.18 | 2.36 | 1.76 |
| Median | 1.58 | 1.64 | 1.89 | 1.56 |
| CV (%) | 9.41 | 9.05 | 9.20 | 7.15 |

| Tibialis anterior |  |  |  |  |
| --- | --- | --- | --- | --- |
|  | Start of sitting | 60 min | 120 min | After leg raise |
| Mean | 2.15 | 2.21 | 2.30 | 2.03 |
| SD | 0.26 | 0.24 | 0.24 | 0.22 |
| SE | 0.05 | 0.05 | 0.05 | 0.05 |
| Min | 1.74 | 1.85 | 1.87 | 1.49 |
| Max | 2.72 | 2.74 | 2.78 | 2.40 |
| Median | 2.09 | 2.20 | 2.32 | 2.03 |
| CV (%) | 12.12 | 11.00 | 10.43 | 10.92 |

Unit: m/s, SD: standard deviation, Min: minimum, Max: maximum, CV: coefficient of variation
